# Exploring biological network structure with clustered random networks

**DOI:** 10.1186/1471-2105-10-405

**Published:** 2009-12-09

**Authors:** Shweta Bansal, Shashank Khandelwal, Lauren Ancel Meyers

**Affiliations:** 1 Center for Infectious Disease Dynamics, Penn State University, University Park, PA 16802, USA; 2 Fogarty International Center, National Institutes of Health, Bethesda, MD 20892, USA; 3 Section of Integrative Biology, University of Texas at Austin, Austin, TX 78712, USA; 4 External Faculty, Santa Fe Institute, Santa Fe, NM 87501, USA

## Abstract

**Background:**

Complex biological systems are often modeled as networks of interacting units. Networks of biochemical interactions among proteins, epidemiological contacts among hosts, and trophic interactions in ecosystems, to name a few, have provided useful insights into the dynamical processes that shape and traverse these systems. The degrees of nodes (numbers of interactions) and the extent of clustering (the tendency for a set of three nodes to be interconnected) are two of many well-studied network properties that can fundamentally shape a system. Disentangling the interdependent effects of the various network properties, however, can be difficult. Simple network models can help us quantify the structure of empirical networked systems and understand the impact of various topological properties on dynamics.

**Results:**

Here we develop and implement a new Markov chain simulation algorithm to generate simple, connected random graphs that have a specified degree sequence and level of clustering, but are random in all other respects. The implementation of the algorithm (ClustRNet: Clustered Random Networks) provides the generation of random graphs optimized according to a local or global, and relative or absolute measure of clustering. We compare our algorithm to other similar methods and show that ours more successfully produces desired network characteristics.

Finding appropriate null models is crucial in bioinformatics research, and is often difficult, particularly for biological networks. As we demonstrate, the networks generated by ClustRNet can serve as random controls when investigating the impacts of complex network features beyond the byproduct of degree and clustering in empirical networks.

**Conclusion:**

ClustRNet generates ensembles of graphs of specified edge structure and clustering. These graphs allow for systematic study of the impacts of connectivity and redundancies on network function and dynamics. This process is a key step in unraveling the functional consequences of the structural properties of empirical biological systems and uncovering the mechanisms that drive these systems.

## Background

Over the last decade, network models have advanced our understanding of biology at all scales, from gene regulatory networks to metabolic cycles to global food webs [1-4]. They are also driving the forefront of sociology, information technology and many other disciplines [5-7]. Researchers often build network models from empirical data and then seek to characterize and explain non-trivial structural properties such as heavy-tail degree distributions, clustering, short average path lengths, degree correlations and community structure [1,6-12]. Many of these properties appear in diverse natural and man-made systems, and can fundamentally influence dynamical processes of and on these networks [13-19].

**Figure 1:**
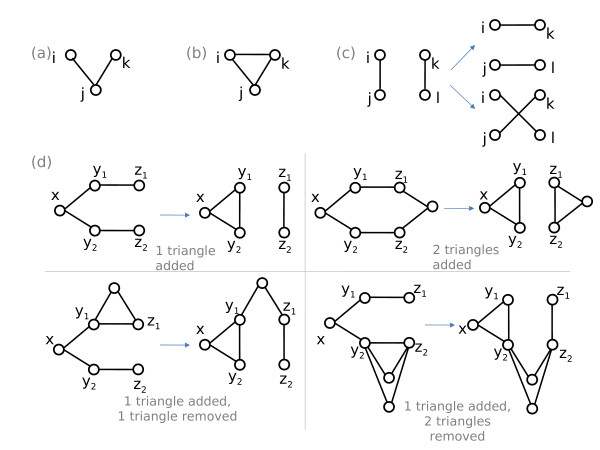
**(a) a triple among the nodes *i, j, k *(b) a triangle among the nodes *i, j, k *(c) A rewiring of edges (*i, j*) and (*k, l*) can result in (*i, k*) and (*j, l*), or (*i, l*) and *j, k*) (d) Four (among many) scenarios for the result of one rewiring step of our algorithm**. The configuration of edges before (left) and after (right) a rewiring step are shown for each scenario. The two bottom scenarios would be rejected by our algorithm as they do not strictly increase the number of triangles.

Clustering is a network characteristic describing the presence of triangles in a network, that is, the propensity of neighbors of a common vertex to also be neighbors with each other. (See Figure 1a and 1b.) It is an important topological characteristic that can significantly impact dynamical processes over complex networks [1,20-23,19]. Clustering is often correlated with local graph properties such as correlations in the number of edges emanating from neighboring vertices [21] and graph motifs [24,4], as well as global properties such as community structure [25].

Clustering in biological and other empirical networks can stem from two sources: (a) it can arise as a byproduct of other, more fundamental, topological properties such as the degree sequence (distribution) or degree correlations (the dependence of a node's degree on its neighbors' degrees); or (b) it can be generated directly by some inherent property or mechanism within the system, for example, "the friends of my friends tend to become my friends" in social networks.

Some researchers have claimed that high clustering is a general feature of complex networks [21]. When we measure clustering in a variety of empirical networks, however, we find that it varies considerably. Table 1 shows that the clustering coefficients and transitivity values (a local and global measure of clustering, respectively) for these networks span the entire range of possible values (zero to one). Thus, it is important to understand not only the origins of clustering, but also the impact of clustering on network functions and dynamics. Towards this end, we introduce a method for generating random networks with a specified level of clustering.

**Table 1:** Topological properties of some empirical networks

Empirical Network	*N*	<*d *>	<*d*^2 ^>	*C*	*T*		
Little Rock Foodweb Interactions	183	27.3	1215	0.37	0.37	0.44	0.58
Yeast Protein Interactions	4713	6.3	152	0.13	0.06	0.14	0.18
*C. elegans *Metabolic Interactions	453	8.9	358	0.66	0.12	0.74	0.60
Vancouver Epidemiological Contacts	2627	13.9	265	0.07	0.09	0.09	0.14
US Air Traffic Links	165	38.0	2765	0.86	0.58	0.97	0.96

The number of nodes (*N*), the average node degree (<*d *>), the mean-squared of node degree (<*d*^2 ^>), clustering coefficient (*C*), transitivity (*T*), Soffer-Vasquez clustering coefficient (), and Soffer-Vasquez transitivity () for a set of empirical networks.

### Related Work

Random graphs are graphs that are generated by some random process [26]. They are widely used as models of complex networks [5] and can assume various levels of complexity. The simplest model for generating random graphs, with only a single parameter, is the Bernoulli or Erdös-Renyi random graph model, which produces graphs that are completely defined by their average degree and are random in all other respects. A slightly more complex and general model is one that generates graphs with a specified degree distribution (or degree sequence) and ones which are random in all other respects [27]. These models can be extended to include additional structural constraints, such as degree correlations or the density of triangles or longer cycles, as we will demonstrate below.

Existing methods for generating clustered graphs, however, do not take this approach. One of the first examples is the seminal work of Watts and Strogatz [1]. They introduced a model that produces networks with high clustering and low average path length (typical distances between pairs of nodes in the network are small), now known as the *small world property*. Although not intended as a generative algorithm for clustered graphs, the model produces graphs with clustering spanning the range from 0 to 1. The graphs generated under this model, however, have rigid spatial structure and cannot accommodate varying degree distributions.

The first algorithms that were designed to generate graphs with a specified level of clustering for arbitrary degree distributions belonged to the class of projected bipartite graphs. Newman [20] introduced a three-step method that first builds a bipartite graph of individuals and affiliations, then projects the bipartite graph to a unipartite graph of individuals only, and finally runs a percolation process over the unipartite graph. This results in a clustered graph with a degree distribution that depends on the original distributions of numbers of individuals per group and groups per individual. The level of clustering in the final graph varies smoothly from 0 to 1 as a function of the percolation probability. In [28], Guillaume suggested a similar bipartite graph approach. Although these approaches can generate clustered graphs with diverse degree distributions, they lack straightforward methods for choosing parameters that yield graphs with not only a pre-specified clustering coefficient but also a pre-specified degree distribution. These algorithms also tends to produce graphs that leave a significant proportion of the graph vertices isolated.

A second class of clustered graph models use "growing network" algorithms [29-31]. The inputs to these models are a degree distribution and level of clustering. The method begins with a set of vertices with no edges; the graph is then "grown" by adding edges based on the degree and clustering constraints. Although the algorithms of this class allow for arbitrary degree distributions and levels of clustering, they either require a complex implementation [29], produce graphs of a highly specific structure [31] or introduce large amounts of degree correlations [31,30].

Finally, the family of statistical models known as exponential random graph (ERG) models [32,33] also provide tools to fit the structure of observed networks, for statistics such as degree distribution and number of triangles. These ERG model-based methods, although they have advanced significantly in recent years (e.g. [34]), still suffer from problems of degeneracy and computational intractability for large networks.

### Our Approach

Here, we present a model that generates undirected, simple and connected graphs with prescribed degree sequences and a specified frequency of triangles, while maintaining a graph structure that is as random (uncorrelated) as possible. (A *simple *graph is one which contains no self-loops (edges from a node to itself) or multiedges (multiple edges between the same pair of nodes); and a *connected *graph is one where every node in the graph is reachable by a path of edges from every other graph node.) Prior models in this area were intended to generate clustered graphs that replicate the properties of real-world networks; our goal, on the other hand, is to generate a class of null networks with arbitrary degree distributions that are simple and connected and have a high density of triangles, but are random in all other respects.

This method thus leads to two valuable applications. First, network structure fundamentally influences the functions of and dynamical processes on networks. We can use clustered random graphs to systematically study the consequences of clustering, both independently and in combination with various degree patterns. Second, these networks can serve as null models for detecting whether an empirical network can be boiled down to its degree distribution and clustering values or, instead, contains substantial degree correlations or other important structures (beyond the byproducts of the degree distribution and clustering). One would first use the algorithm to generate an ensemble of networks that match the empirical degree sequences and clustering values, and then compare the structural, functional, or dynamical properties of the empirical network to those of the clustered random networks. We focus here on the role of these networks as null models as it is crucial to have appropriate random controls in the study of biological systems, as has been demonstrated in [24,35,36].

The rest of this article is organized as follows. In the Implementation section, we review common measures of clustering and introduce our Markov chain model and algorithm for generating clustered graphs with a specified degree sequence. In the Results section, we test our algorithm with numerical simulations and explore the structural properties of the generated graphs. The Discussion section is devoted to a demonstration of the randomly generated clustered networks as null networks for the analysis of empirical networks. We finish off with our conclusions, presenting the benefits of our Markov Chain simulation method for biological networks.

## Implementation

Our clustered random graph generation method begins with a random graph and iteratively rewires edges to introduce triangles. Network rewiring, also known as edge swapping, is a well-known method for generating networks with desired properties [37,36,38]. Two edges are called *adjacent *if they connect to a common node. Each *rewiring *is performed on two non-adjacent edges of the graph and consists of removing these two edges and replacing them with another pair of edges. Specifically, a pair of edges (*i, j*) and (*k, l*) is replaced with either (*i, k*) and (*j, l*), or (*i, l*) and (*j, k*) (as illustrated in Figure 1c). This change in the graph leaves the degrees of the participating nodes unchanged, thus maintaining the specified degree sequence. Below we describe a rewiring algorithm that increases the level of clustering in a random graph, while preserving the degree sequence.

The algorithm we develop below is implemented in Python as ClustRNet. It is based on Networkx, an open-source Python library available for download at [39], which provides standard graph library functionality (e.g. data structure, input/output, and layouts). The source code for ClustRNet, along with documentation and test network datasets, is available on the web [40]. Our algorithm joins a existing suite of random graph model-based software tools for the analysis of biological networks and the dynamics on them [41,42].

### Measures of Clustering

We begin with a graph *G *= (*V, E*) which is undirected and simple. *V *is the set of vertices of *G *and *E *is the set of the edges. We let *N *= |*V*| and *M *= |*E*| denote the number of nodes and edges in *G*, respectively. The *degree *of a node *i *will be denoted *d*_*i*_. The set of degrees for all nodes in the graph makes up the *degree sequence*, which follows a probability distribution called the *degree distribution*.

Clustering is the likelihood that two neighbors of a given node are themselves connected. In topological terms, clustering measures the density of *triangles *in the graph, where a triangle is the existence of the set of edges (*i, j*), (*i, k*), (*j, k*) between any triplet of nodes *i, j, k *(Figure 1b).

To quantify the local presence of triangles, *δ*(*i*) is defined as the number of triangles in which node *i *participates. Since each triangle consists of three nodes, it is counted thrice when we sum *δ*(*i*) for each node in the graph. Thus the total number of triangles in the graph is

A *triple *is a set of three nodes, *i, j, k *that are connected by edges (*i, j*) and (*i, k*), regardless of the existence of the edge (*j, k*) (Figure 1a). The number of triples of node *i *is simply

assuming *d*_*i *_≥ 2. To compute the total number of triples in the graph, *τ*(*G*), we sum *τ*(*i*) for all *i *∈ *V*.

The *clustering coefficient *was introduced by Watts and Strogatz [1] as a local measure of triadic closure. For a node *i *with *d*_*i *_≥ 2, the clustering coefficient *c*(*i*) is the fraction of triples for node *i *which are closed, and can be measured as *δ*(*i*) = *τ*(*i*). The clustering coefficient of the graph is then given by:

where *N*_2 _is the number of nodes with *c*(*i*) ≥ 0. Some authors do define the clustering coefficient for all nodes of *G *[43].

A more global measure of the presence of triangles is called the *transitivity *of graph *G *and is defined as:

Although they are often similar, *T*(*G*) and *C*(*G*) can vary by orders of magnitude [22]. They differ most when the triangles are heterogeneously distributed in the graph.

These traditional measures of clustering are degree-dependent and thus can be biased by the degree sequence of the network. The maximum number of possible triangles for a given node *i *is just its number of triples (*τ*(*i*)). For a node which is connected to only low degree neighbors, however, the maximum number of possible triangles may be much smaller than *τ*(*i*). To account for this, a new measure for clustering was introduced in [22] that calculates triadic closure as a function of degree and neighbor degree. Specifically, the Soffer-Vasquez clustering coefficient () and transitivity () are given by:

where *ω*(*i*) measures the number of *possible *triangles for node *i*, and *N*_*ω *_is the number of nodes in *G *for which *ω*(*i*) > 0. We note that  and  are undefined if *ω*(*G*) = Σ_*i *_*ω*(*i*) = 0. *ω*(*i*) is computed by counting the maximum number of edges that can be drawn among the *d*_*i *_neighbors of a node *i*, given the degree sequence of *i*'s neighbors; this value is often smaller than [22]. For example, consider a star network of five nodes, where four nodes have degree 1 and one node has degree 4. Although the total number of triples is *τ*(*G*) = 6, the number of possible triangles is *ω*(*G*) = 0 because the degree one nodes preclude their formation. The computation of *ω*(*i*) must be done algorithmically and is not possible in closed form. (From here on, we refer to  as the SV-clustering coefficient and to  as the SV-transitivity.)

### Generative Model

Here we develop a model to generate a simply connected random graph with a specified degree sequence and a desired level of clustering. Generating random graphs uniformly from the set of simply connected graphs with a prescribed degree sequence is a well-studied problem with algorithmic solutions [37]. One of the simplest and most popular of these generative algorithms was suggested by Molloy and Reed and is known as the configuration model [27]. Given a specific realizable degree sequence [44], {*d*_*i*_}, this method assigns *d*_*j *_half-edges to each node *j*, and then randomly connects pairs half-edges to create edges until there are no half-edges left. (A *realizable *degree sequence is one which satisfies the Handshake Theorem (the requirement that the sum of the degrees be even) and the Erdos-Gallai criterion (which requires that for each subset of the *k *highest degree nodes, the degrees of these nodes can be "absorbed" within the subset and the remaining degrees.) Although the model sometimes produces graphs that are not simple or connected, this can be remedied by subsequently removing multiple edges and self loops from the constructed graph and keeping only the largest connected component [37]. Our method begins by using this approach to generate a simple, connected random graph *G*, with a specific realizable degree sequence *D*. We then introduce triangles into *G *using a Markov Chain process without disturbing the degree sequence until we achieve the desired level of clustering, as follows.

Let *G*_*D *_be the set of all simple, connected graphs with degree sequence *D*. If  are the graphs of *G*_*D*_, then we let  be the states of the Markov chain, *P*, where *X*_*i *_represents the state in which our graph *G *= *G*_*i*_. The states *X*_*i *_and *X*_*i*+1 _are connected in the Markov Chain if *G*_*i *_can be changed to *G*_*i*+1 _with the rewiring of one pair of edges. The state space of the Markov chain *P *is connected because there exists a path from *X*_*i *_to *X*_*j *_(for any pair *i, j*) by one or more rewiring moves that leave the degree sequence unchanged [45].

Our clustered graph generation algorithm involves starting with the random graph *G *(generated with the configuration model above) and transitioning from the state corresponding to *G *(*X*_*G*_) to other states of *P *until a halting condition is reached. A transition from one state of the Markov chain to another only occurs when the algorithm makes an edge rewiring that both increases the clustering of the graph and leaves the graph connected. Since a rewiring does not alter the degree sequence of the graph, the rewired graph is still in *G*_*D*_. The transition probabilities of the Markov chain for a pair of connected states, *X*_*i *_to *X*_*j*_, are:

where *clust*(*G*_*x*_) is a clustering measure for graph *G*_*x*_, which can be replaced by any of the measures introduced in Section. The algorithm continues searching for a feasible rewiring (one that increases the clustering and does not disconnect the graph) until one is found. If a feasible move is not found, a transition is not made and the process remains in the current state.

The Markov chain above is finite and aperiodic, but not irreducible as the process can never transition to a state in which the graph has lower clustering. It does, however, have an absorbing state, X_*_, in which the transitivity of G_* _is greater than or equal to the desired transitivity or is the maximum possible transitivity given the particular degree sequence and connectivity constraints.

### Algorithm

To generate clustered graphs, we apply the above Markov Chain simulation model by iteratively applying rewirings that increase graph clustering. Each rewiring takes a set of five nodes {*x, y*_1_, *y*_2_, *z*_1_, *z*_2_}, connected by four edges {(*x, y*_1_), (*x, y*_2_), (*y*_1_, *z*_1_), (*y*_2_, *z*_2_)}, and swaps the outer edges: {(*x, y*_1_), (*x, y*_2_), (*y*_1_, *y*_2_), (*z*_1_, *z*_2_)}(illustrated in Figure 1d). This introduces a triangle among nodes {*x, y*_1_, and *y*_2_}, without perturbing the degree sequence. The algorithm proceeds as follows:

**Input**: A realizable degree sequence {*d*_*i*_} a desired clustering value, *target*

**Initialization**: Generate a random graph *G *with degree sequence {*d*_*i*_} (using the configuration model), and measure the clustering of *G*, *clust*(*G*).

**while ***clust*(*G*) <*target ***do**

1. uniformly select a random node, *x*, from the

    set of all nodes of *G *such that *d*_*x *_> 1.

2. uniformly select two random neighbors, *y*_1 _

   and *y*_2_, of *x *such that *d*_*y*1 _> 1 and

   *d*_*y*2 _> 1 and *y*_1_≠*y*_2_.

3. uniformly select a random neighbor, *z*_1 _

   of *y*_1 _and a random neighbor, *z*_2 _of

   *y*_2 _such that *z*_1 _≠ *x*, *z*_2 _≠ *x*,

   *z*_1 _≠ *z*_2_.

4. *G*_*cand*_: = *G *where *G*_*cand *_is the candidate

   graph to which the transition may be made.

5. **if **(*y*_1_, *y*_2_) *and *(*z*_1_, *z*_2_) *do not exist ***then**

   Rewire two edges of *G*_*cand*_: delete (*y*_1_, *z*_1_) and (*y*_2_, *z*_2_), add (*y*_1_, *y*_2_) and (*z*_1_, *z*_2_).

   **end**

6. Update the value of *clust*(*G*_*cand*_) by measuring

*   δ *(*i*) (and *ω *(*i*) if relevant) for the nodes involved

   in the rewiring and their neighbors.

7. **if ***clust*(*G*_*cand*_) > *clust*(*G*) *and G*_*cand *_

   *is connected ***then**

   *G*: = *G*_*cand*_

   **end**

end

**Output**: A random graph, *G *with degree sequence {*d*_*i*_} and *clust*(*G*) ≥ *target*.

The algorithm terminates when the graph attains at least the desired level of clustering or reaches a threshold number of unsuccessful rewiring attempts. In the latter case, the algorithm returns the graph with the maximum clustering achieved. For practical purposes, a threshold is placed on the number of unsuccessful attempts made by the algorithm in ClustRNet for the case that the desired clustering cannot be reached. Due to the random restarts made at every step, the algorithm is prevented from getting trapped in local minima.

The algorithm is designed to increase clustering while preserving both the degree sequence and connectedness of the graph. However, there are some cases where the desired clustering can only be reached by disconnecting the graph; and thus ClustRNet provides the option of removing the connectivity constraint (see Additional file 1, Figure S2).

#### Choice of Clustering Measure

The algorithm is defined independent of the choice of clustering measure. The term *clust*(*G*) in the algorithm above can be replaced by any clustering measure described in Section. ClustRNet includes all four of these clustering measures (*C*, , *T*; ).

The algorithm output varies with the choice of clustering measure. The clustering coefficient is a local measure; and thus *C *and  yield networks that are only locally optimized for the desired level of clustering. The algorithm may have difficulty attaining target clustering values when using the absolute clustering measures (*C *or *T*) because of joint degree constraints (the degrees of adjacent nodes) on the possible numbers of triangles, as with the example presented in Section. The Soffer-Vasquez clustering measures, which explicitly consider joint degree constraints, provide a way around this difficulty [22]. Although the rewiring in our algorithm changes the joint degree distribution (and thus the degree correlations) of the graph, *ω*(*G*) is not altered significantly during network generation (as shown in Additional file 1, Figure S3). Thus, when using  or , clustering is increased primarily by the addition of triangles (that is, increasing *δ *(*G*)) rather than decreasing *ω*(*G*)).

#### Types of Graph Changes

**Figure 2:**
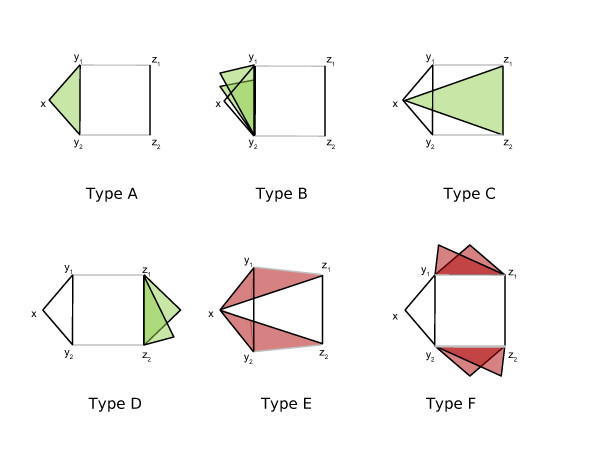
**Possible triangle additions (green) and removals (red) in one step of the rewiring procedure**. Black lines represent existing edges and edges added after a rewiring event, gray lines represent edges lost during a rewiring event.

As shown in Figure 2, there are six types of triangles that can be added or removed for every pair of edges that are rewired. As illustrated in Figure 1d, these additions and removals can occur in combination.

• Type A: The addition of the edge between vertices *y*_1 _and *y*_2 _guarantees the addition of one triangle in every rewiring event.

• Type B: The addition of the edge (*y*_1_, *y*_2_) could create new triangles with shared neighbors of *y*_1 _and *y*_2_.

• Type C: The addition of the edge (*z*_1_, *z*_2_) could add a triangle if there existed edges between *x *and *z*_1 _and *x *and *z*_2_.

• Type D: The addition of the edge between vertices *z*_1 _and *z*_2 _could create new triangles with shared neighbors of *z*_1 _and *z*_2_.

• Type E: The removal of edges (*y*_1_, *z*_1_) and (*y*_2_, *z*_2_) removes one triangle each if the edges (*x, z*_1_) or (*x, z*_2_) exist.

• Type F: The removal of the edges between vertices *y*_1 _and *z*_1_, and *y*_2 _and *z*_2 _could lead to the removal of existing triangles with shared neighbors of *y*_1 _and *z*_1 _or *y*_2 _and *z*_2_.

We note that although the type A addition is a special case of type B, the type C addition is a special case of type D, and the type E removals are a special case of type F, we distinguish them because they have different probabilities of occurrence. Our look-ahead strategy only allows rewiring moves when the total number of Type E and F losses is fewer than the total number of Type A, B, C, and D gains.

### Computational Complexity

Like many heuristic search methods, the algorithm we propose can be computationally expensive. The method outlined in Section 2.2 requires *O*(*M*) steps to generate a connected graph, and up to *O*(*M*) steps to randomize the graph, where *M *is the number of edges in the graph. At each step of randomization, we test that the graph remains connected (an *O*(*M*) operation), resulting in an overall *O*(*M*^2^) random network generation process. A naive computation of the transitivity/clustering coefficient requires checking every node for the existence of edges between every pair of neighbors of the node. This step requires *O*() operations, where *N *is the number of nodes and *d*_*max *_is the maximum degree of any node in the graph. The most expensive step of our algorithm is the introduction of triangles via rewiring. A single rewiring step requires *O*(*M*) operations for switching edges, checking for connectivity and updating the clustering measure. Although we cannot analytically calculate the number of attempted rewiring steps required to reach the desired transitivity, we have found it empirically to be *O*(*M*). Thus, the average complexity of the clustered network algorithm presented here is *O*(*M*^2^). This complexity has been computed for the most naive versions of our algorithms; and more efficient implementations may improve the complexity greatly. For example, we might improve efficiency by performing connectivity tests once every *x *rewirings (for some number *x*) rather than during every rewiring, as proposed in [46].

## Results

### Performance

**Figure 3:**
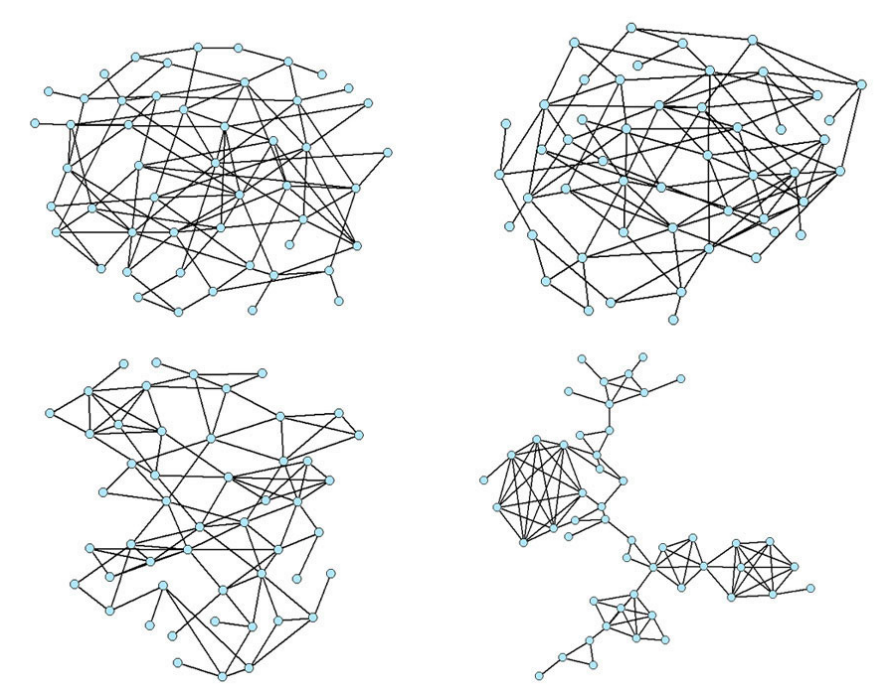
**The evolution with our algorithm of a Poisson-distributed random graph with 50 nodes from (a)  ≈ 0,(b)  = 0.1,(c)  = 0.5 and (d)  = 0.8, with the connectivity constraint**.

To test our algorithm, we generate networks with three different degree distributions and for a range of clustering target values. Specifically, we use Poisson (*p*_*d *_= *e*^-*λ *^*λ*^*d*^/*d*!), exponential (*p*_*d *_= (1 - *e*^*κ*^)*e*^-*κ*(*d*-1)^) and a truncated scale-free (*p*_*d *_= *d*^-*γ*^*e*^-*d*/*κ*^/*Li*_*γ*_(*e*^-1/*κ*^)) degree distribution, each with a mean degree of five. Starting with random graphs with specific degree sequences matching these degree distributions, we rewire the networks towards (1) SV-transitivity (()) targets and (2) transitivity (*T*) targets in addition to allowing the algorithm to generate disconnected graphs. These targets allow us to evaluate how the clustering measure and connectivity requirement constrain the results, and the second target, in particular, allows us to compare results to other algorithms. Figure 3 illustrates the rewiring of a network with a Poisson distributed degree sequence evolving towards higher transitivity.

**Figure 4:**
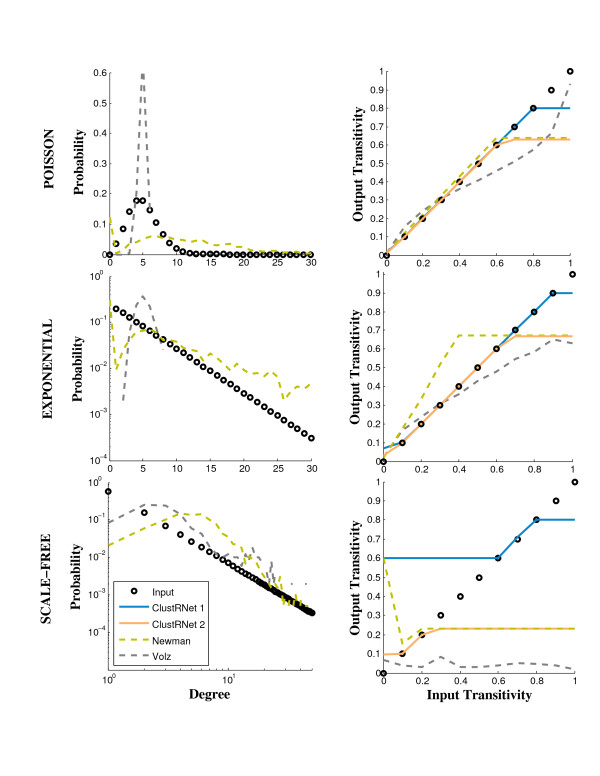
**Discrepancies between input and average output degree distributions (left panels) and average transitivity values (right panels) for an ensemble of 15 Poisson (top panels), exponential (middle panels) and scale-free graphs (bottom panels) as generated by our algorithm and the algorithms presented in **[30]**and **[20]. Each graph has *N *= 500 and mean degree, ⟨*d*⟩ = 5. In the left graphs, the input degree distribution is shown as a black circles; and output degree distributions are shown for the Newman (green dashed line) and the Volz (gray dashed line) algorithms. Output degree distributions are not shown for ClustRNet as the degree sequence always perfectly match the input. In the right graphs, the input is shown as black circles, and output transitivity values are shown for two runs: (1) using SV-transitivity (()) as the clustering measure in ClustRNet (blue line), and (2) ClustRNet [without a connectivity constraint] (orange line), the Newman algorithm (green dashed line) and the Volz algorithm (gray dashed line), all with transitivity (()) as the clustering measure.

We evaluate the performance of our algorithm in comparison to one representative network growth algorithm [30] and one representative bipartite network method [20]. Specifically, we measured the discrepancies between input and output degree distributions (Figure 4 left graphs) and transitivity values (Figure 4, right graphs). Our algorithm preserves the input degree sequence perfectly, while there are considerable mismatches between the input and output degree distributions in the Volz and Newman models. For both comparisons, the transitivity values of the output graphs from our algorithm exactly match the target transitivity values, when those values can be attained given the network topology and the requirements of the algorithm. Some values at the lower end of the clustering scales cannot be reached because the expected transitivity for random graphs of specified degree distributions scales as  where *p*_*k *_is the degree distribution [21,8,43]. This value is small for the Poisson degree distribution but can be quite high (especially when measured as SV-transitivity) for highly-skewed degree distributions such as the scale-free degree distribution. For the first comparison, the connectivity constraint imposes a maximum on the attainable clustering value, thus the highest SV-transitivity values cannot be reached without disconnecting the graphs. In these cases, our algorithm returns the graph with the largest attainable SV-transitivity that is less than the desired SV-transitivity. For the second comparison, (with requirements to match the other algorithms), our algorithm performs better in all cases compared to the Volz and Newman models. Due to the definition of the standard transitivity measure (*T*), however, we see that the networks reach a maximum *T *value, beyond which no further clustering can be accommodated by the network topology.

### Structural Properties of Generated Networks

There are several other topological properties (besides degree sequence and clustering) that can strongly influence network function and dynamics. Among these are degree correlations (the dependence of a node's degree on its neighbors' degrees), community structure (groups of nodes that are highly intra-connected and only loosely inter-connected), and average path length (typical distances between pairs of nodes in the network). We have specifically developed this model to increase clustering with minimal structural byproducts. Thus, we confirm that we have reached this goal by measuring the above properties in the networks generated by our algorithm.

**Figure 5:**
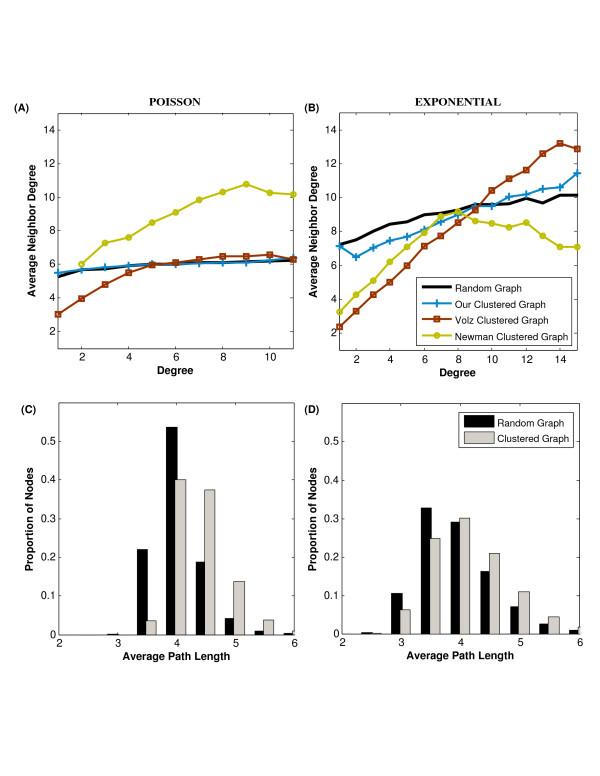
**Degree correlations (A and B) and average path lengths (C and D) in random graphs with specified degree distributions (Poisson and exponential with mean degree = 5) compared to clustered random graphs with the same degree distributions and *T *= 0.5 generated by our algorithm (with the connectivity constraint), as well as the Volz **[30]**and Newman **[20]**algorithms (in A and B)**. The graphs present averages over 15 graphs generated by each algorithm. Our algorithm introduces fewer degree correlations than the alternatives, and the clustered graphs have only slightly higher average path lengths than their random counterparts: 4.05 for the Poisson random graphs versus 4.39 for the clustered graphs; and 3.95 for the exponential random graphs versus 4.14 for the clustered graphs.

We evaluated the extent to which the algorithm introduces degree correlations by comparing random (unclustered) graphs to clustered random graphs generated by our algorithm and the Volz [30] and Newman [20] algorithms (Figure 5. While our algorithm essentially preserves the correlation structure of the random graph, the other algorithms produce highly correlated graphs. Results are not shown for scale-free graphs as initial transitivity values were larger than 0.5 for all generated graphs.

Several authors have discussed the relationship between clustering and community structure [8,25,47,21]. As Figure 3 shows, the addition of triangles leads to modular structure. This behavior is not surprising: as the number of edges in the graph is constrained, sets of connected nodes with high *ω*(*i*) values (often high-degree nodes) must be brought together to create additional clustering. Although the presence of a significant proportion of triangles tends to separate the network into modules, it is not clear that clustering is always sufficient to explain the modular structure of a graph. We explore this further below.

Short average path lengths are a characteristic feature of random graphs [26]. To quantify the impact of our algorithm on path lengths, we calculated the average path length for each node to all other (*N *- 1) nodes, and then compared the distributions of these values for several random and random clustered graphs (Figure 5). While our algorithm mostly maintains short average path lengths, the mean of the path length distribution does tend to be slightly larger for the clustered graphs than for the corresponding random graphs. The intuition behind this increase in average path length may lie in the increased community structure: as graphs become more clustered and separate into subgroups, nodes in different groups require more links to reach each other (Figure 3). Given that our algorithm can generate graphs of high clustering while preserving short path lengths, this introduces a novel method of generating graphs with the small world property without the correlations of Watts-Strogatz graphs [1].

## Discussion

### Application: Analysis of Empirical Networks

It is crucial to have random controls in the study of biological systems. Our algorithm can be used to generate null models and applied to the detection of structure in empirical biological networks. We can generate ensembles of clustered random networks with empirically estimated degree sequences and clustering values to ascertain whether empirical networks have significant non-random structure in other respects. We demonstrate this application using representatives from four classes of biological networks. We also analyze one non-biological network that is made of human transportation links as it provides contrast to the range of topological properties and design principles found in the biologically-motivated networks. The five real networks are as follows: a) a trophic exchange network for the Little Rock Lake in Wisconsin [48]; b) a protein interaction network for yeast [3]; c) a metabolic network for the eukaryote *Caenorhabditis elegans *[49]; d) a network made up of epidemiologically-relevant contacts for individuals in the city of Vancouver [13]; and e) a transportation network, made up of US metropolitan areas connected by air travel [50]. These networks represent a diverse set of applications and are systems that are well-studied in their respective literatures. The basic statistics of these networks, including clustering values, are listed in Table 1.

We use the following method to quantify deviations from randomness in these networks. First, we use our algorithm to generate 25 clustered random networks constrained to match the empirical degree sequence and clustering values. Second, we select a set of network topological measures (other than degree distribution and clustering), and compare these quantities for the empirical graph to the corresponding average quantities across the ensemble of generated graphs.

Specifically, we generate 25 clustered random networks for each empirical network, constrained to match the empirical degree sequence and SV-transitivity. In addition to the degree and clustering metrics, we also calculated diameter (longest shortest path length between any pair of nodes in the graph) [51], degree correlation coefficient [11] and modularity (degree of community structure) [52] (Table 2). Other than diameter, each of these metrics range from 0 to 1. The standard deviations for all statistics are negligible across the ensembles and thus not reported. For every statistic, we also give the deviation between the empirical value and the average across the generated ensemble of random clustered networks (specifically, deviation = ensemble mean - observed value). Small deviations suggest that the empirical network structure boils down to the degree distribution and clustering, and thus we turn our attention to possible mechanisms underlying these properties. In contrast, large deviations suggest that there are other fundamental properties to consider in addition to or, perhaps, instead of clustering.

**Table 2:** Comparisons between empirical networks and clustered random networks

Generated Network Type	*N*	<*d *>	<*d*^2 ^>	*T*		*Diam*	*r*	*Q*
Little Rock Foodweb Interactions	183	27.3	1215	0.38 [0.009]	0.58 [0.0]	4 [0.0]	-0.09 [0.15]	0.11 [-0.21]
Yeast Protein Interactions	4713	6.3	152	0.07 [0.01]	0.18 [0]	12.5 [0.5]	0.11 [0.38]	0.39 [-0.10]
*C. elegans* Metabolic Interactions	453	8.9	358	0.14 [0.02]	0.60 [0]	6 [-1]	-0.19 [0.04]	0.29 [-0.09]
Vancouver Epidemiological Contacts	2627	13.9	265	0.09 [0]	0.14 [0]	6 [0]	0.15 [-0.4]	0.28 [-0.15]
US Air Traffic Links	165	38.0	2765	0.58 [0]	0.97 [0]	3 [0]	-0.55 [0]	0.11 [-0.01]

For each empirical network, we generated 25 random graphs constrained to have the observed degree sequences and Soffer-Vasquez transitivity values. The table reports average values of several network statistics for the clustered random graphs: network size (*N*), mean degree (⟨*d*⟩), mean squared degree (⟨*d*^2^⟩), Soffer-Vasquez clustering coefficient (), Soffer-Vasquez transitivity (), maximum shortest path length between any two nodes (*diam*), degree correlation coefficient (*r*), and modularity (*Q*). The value given in brackets is the deviation of the ensemble mean from the corresponding statistic for the empirical network. (A positive deviation indicates that the ensemble mean was greater than the empirical statistic and vice versa.) Deviations are not listed for *N*, ⟨*d*⟩ and ⟨*d*^2^⟩ as network size and degree sequence are constrained by our algorithm to match the empirical networks perfectly.

Of all the empirical networks analyzed, the random counterparts of the the US air traffic network are the only ones that have structural properties almost identical to the real network (with the network of Vancouver epidemiological contacts being the next closest). This suggests that the structure of the US air traffic network comes almost exclusively from its degree patterns. (In fact, even the high clustering is explained exclusively by the degree patterns.) We note that the US air traffic network is the only non-biological one and the most engineered of the networks we consider, and thus may have fewer emergent properties. The remaining empirical networks (all biological) differ considerably from their random counterparts, suggesting that there are important mechanistic features not captured in the random model.

Degree correlations vary somewhat systematically among the four biological networks (Table 2). The Vancouver human epidemiological contact network has significantly higher degree assortativity than our random networks, thus showing that the positive degree correlations are not just the result of degree distribution or clustering, both of which have been found to be positively correlated with assortativity [53]. This suggests the existence of social rules among humans that go beyond (a) variation in numbers of "friends" and (b) the tendency for "my friend's friend also to be my friend" [11]. The remaining biological networks (the yeast protein interactions, the Little Rock Lake foodweb, and the *C. elegans *metabolic networks), on the other hand, all have negative degree correlations. Our results show that the *C. elegans *metabolic network, in particular, has degree correlations approximately equal to the amount expected to arise as a random byproduct of degree distribution and clustering. One reason that a biological network only show random degree correlations might be due to the lack of a clear functional or structural advantage for strong correlations: negatively correlated networks are vulnerable to failures because functionality often depends on a few high degree nodes that provide essential connectivity. If any of these fail (e.g., because of a gene deletion in a metabolic network) the whole system fails [11,12]. On the other hand, positively correlated networks, which have short distances between hub (high-degree) nodes, may be less favorable because they allow for the propagation of random perturbations (e.g., changes in the concentration of a protein in a protein-interaction network) [36].

All of the natural networks we study have significantly higher modularity than the corresponding clustered random networks, despite having a wide range of transitivity values. This suggests that clustering and community structure are not necessarily positively correlated, as has been previously suggested [52,8]. The high modularity of the Little Rock foodweb, in particular, has been attributed to its high clustering [54]. Our generated clustered random graphs, however, indicate that the degree distribution and high transitivity only account for about half the modularity of the foodweb graph (Table 2). There is an extensive literature on the presence and evolution of modularity in protein, metabolic, and ecological networks highlighting its possible roles in functional specialization, innovation and robustness [55-60]. Since clustering and the mechanisms that give rise to it cannot fully account for the modularity of these empirical networks, such mechanistic explanations for the structure are warranted.

## Conclusions

In this work, we have introduced a Markov chain simulation algorithm to generate clustered random graphs with a specified degree sequence and level of clustering. Our algorithm perfectly preserves the degree sequence of a random graph and generally maintains other fundamental properties of random graphs like short path length and low degree correlations. The use of random graphs as controls is a common and effective method for identifying important structural characteristics of biological networks (as, for example, has been seen in [61,54,62,49,13]). Our method provides a new null model for use with this technique. Since this method is based on a dynamic process, it can be used to generate both static networks with a specified amount of clustering and dynamic networks with evolving levels of clustering. Furthermore, since the process is a "memoryless" one, additional clustering can be added to any network without having to grow a new one from scratch. These clustered networks can provide valuable insights into the interdependent impacts of connectedness and redundancy on biological processes, and serve as appropriate null models for investigating the biological significance of other structural attributes.

## Availability and Requirements

• *Project name: *ClustRNet

• *Project home page: *http://sbansal.com/ClustRNet/

• *Operating system(s): *Platform independent

• *Programming language: *Python 2.5

• *Other requirements: *Networkx Python package 2.5

• *License: *BSD-style

• *Any restrictions to use by non-academics: *None

## Authors' contributions

SB, SK and LAM contributed to algorithm design, implementation and manuscript writing. All authors read and approved the final manuscript.

## Supplementary Material

Additional file 1 **Supplementary analysis**. Additional analysis of algorithm with figures. Click here for file
