# Supplementary material for: Exploring biological network structure with clustered random networks
Source: BMC Bioinformatics. 2009 Dec 9;10:405. doi: 10.1186/1471-2105-10-405 (PMC2801686; doi:10.1186/1471-2105-10-405)
Supplement: Additional file 1 — Supplementary analysis. Additional analysis of algorithm with figures. [file 1471-2105-10-405-S1.PDF]

Exploring Biological Network Structure with Clustered Random Networks: Supplementary Information

Shweta Bansal, Shashank Khandelwal, Lauren Ancel Meyers

Supplemental Figures

We evaluated the effectiveness of an algorithm which accepts all rewirings regardless of their effect on the number of triangles. Recall that our main algorithm only makes rewirings that increase the number of triangles. In Figure S1, we show that the permissive algorithm is not effective in achieving the desired levels of clustering. Additionally, other structural properties of the network, e.g. degree correlations, are significantly altered from the original graph in this case (as shown in the right panel of Figure S1.)

Figure S2 illustrates a network in which disconnection is required to achieve maximal clustering.

Figure S3 shows that our algorithm does not change the number of possible triangles ($\omega(G)$) in the graph drastically.

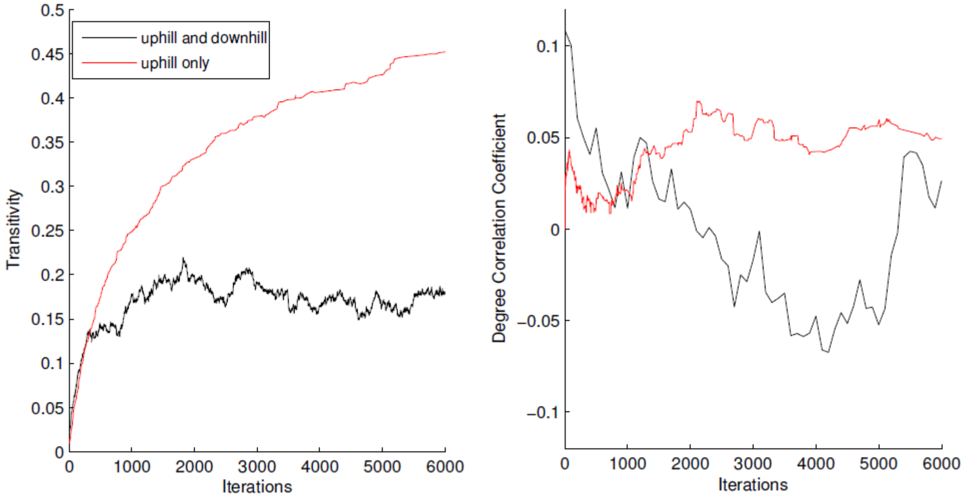

Figure 1: The effect of allowing both uphill (rewirings that increase the total number of triangles) and downhill (rewirings that decrease the total number of triangles) moves. These results are shown for a Poisson distributed graph of 500 nodes. In the left panel, we see that allowing all rewirings is not effective in reaching the desired transitivity ($T = 0.45$). We also find that the structure of the graph is altered significantly in the process of making all rewirings. The degree correlation coefficient, for example, varies significantly with each rewiring (as shown in the right panel.

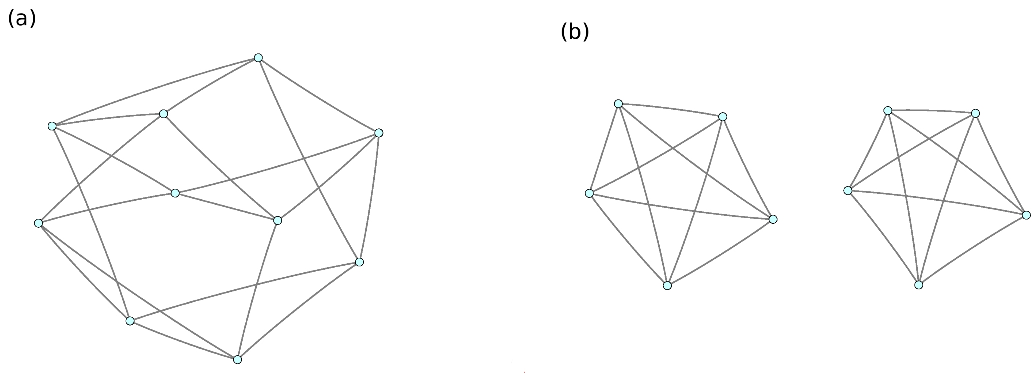

Figure 2: (a) A random graph with 10 nodes, each of degree 4. (b) The graph in (a) must be disconnected to be maximally clustered ($C = T = \tilde{C} = \tilde{T} = 1$).

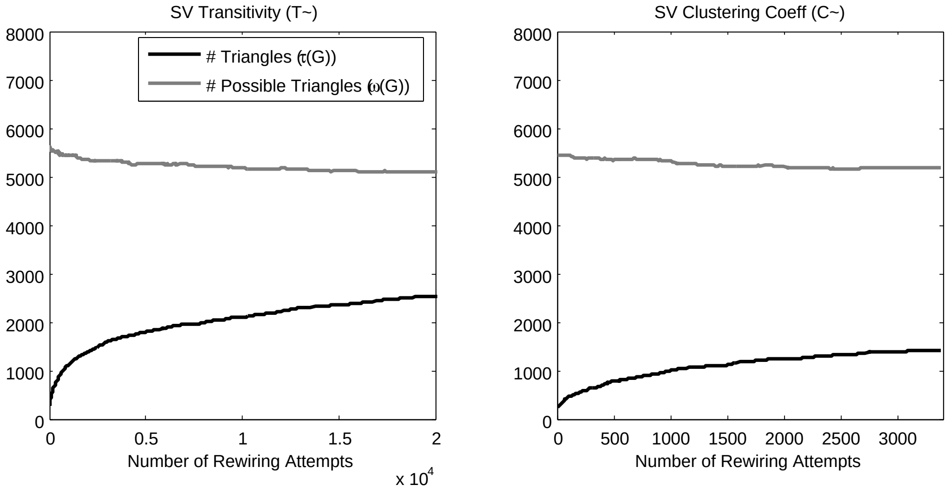

Figure 3: The numbers of triangles $\delta(G)$ and possible triangles $\omega(G)$ in the graph as the algorithm progresses. $\omega(G)$ does not vary substantially during graph generation. These results are for a Poisson distributed graph of 500 nodes, to which triangles are added until reaching (a) Soffer-Vasquez transitivity = 0.5 and (b) Soffer-Vasquez clustering coefficient = 0.5.
